# Comparing Web and Touch Screen Transaction Log Files

**DOI:** 10.2196/jmir.3.2.e18

**Published:** 2001-05-23

**Authors:** David Nicholas, Paul Huntington, Peter Williams

**Affiliations:** ^1^The Internet Studies Research GroupDepartment of Information ScienceCity UniversityLondonUK

**Keywords:** Metrics, access log files, Internet, health, Web site, touch screen kiosks, robust measures, caching

## Abstract

**Background:**

Digital health information is available on a wide variety of platforms including PC-access of the Internet, Wireless Application Protocol phones, CD-ROMs, and touch screen public kiosks. All these platforms record details of user sessions in transaction log files, and there is a growing body of research into the evaluation of this data. However, there is very little research that has examined the problems of comparing the transaction log files of kiosks and the Internet.

**Objectives:**

To provide a first step towards examining the problems of comparing the transaction log files of kiosks and the Internet.

**Methods:**

We studied two platforms: touch screen kiosks and a comparable Web site. For both of these platforms, we examined the menu structure (which affects transaction log file data), the log-file structure, and the metrics derived from log-file records.

**Results:**

We found substantial differences between the generated metrics.

**Conclusions:**

None of the metrics discussed can be regarded as an effective way of comparing the use of kiosks and Web sites. Two metrics stand out as potentially comparable and valuable: the number of user sessions per hour and user penetration of pages.

## Introduction

There are an increasing number of formats by which digital health information can be disseminated. The media that have been employed to disseminate health information since the "digital revolution" include the Internet, CD-ROMs, WAP (Wireless Application Protocol) phones, touch screen public kiosks, videoconferencing, and cable television. One-half of all American homes now have access to the Internet. Britain is said to be leading the European "race" to get online [1]. Accompanying (and fuelling) this online boom is the growing demand to provide the public with informed choices. To give one example, less than one year after Medline became freely available on the Web, the number of searches increased tenfold, with no less than 30% of users being members of the general public [2]. Cyber Dialogue [3] claims that in the United States alone, nearly 41 million Internet users consult the Web for health care information.

While many people have been eagerly watching for the latest Internet development, touch screen digital health information kiosks (and their hybrid forms) have quietly spread around Britain. There are probably more than 200 of them altogether, in surgeries, hospitals, health centers, and shopping centers and even in airports and railway stations. It has been predicted that the number is likely to double over the next couple of years. Kiosks can produce comprehensive and in-depth information and can appeal to people that do not have Internet access at home-for example, the elderly and the poor. Little research has been done, however, to test the public's receptivity to this new medium.

Use is clearly an important characteristic in assessing the popularity of a touch screen kiosk and in making comparisons between Web sites and kiosks. The source of most use data is the digital logs that record user activity on a continuous and real-time basis. The logs provide data on what people have done, not on what people might do or remember having done-this gives the logs their strength, and differentiates them from other data-capture methods, like questionnaires and interviews. There is much demand from sponsors, Web site and kiosk owners, and marketing departments for this information. To meet the demand, a range of metrics has been introduced: pages viewed, time on-line, page view time, and number of users (visitors). These metrics are much bandied about by the press. Terms like hits and visitors have entered our everyday vocabulary.

Surprisingly then, generating kiosk-use metrics from log files has not been well researched, despite the fact that it is important to undertake such studies for a number of reasons.

Firstly, such analyses give information-providers data on, for example, which pages, pieces of information, or subjects are being accessed and to what extent. This data can be cross-tabulated by age and gender. From this information policy decisions can be made regarding increasing, changing, or reducing the information provided, depending on who is targeted to receive the information. To give one example, if a document posted on a kiosk dealing with some aspect of drug abuse was shown to be accessed by few of the target age group (eg, 18-25 year olds), but by many more 40-50 year olds the information provider would be armed with information indicating that it is the older age group (possibly parents of teenagers) who read pages on this topic. The page could thus be modified either to provide more information that may be relevant to parents, or to repackage the information in another attempt to reach the original intended target.

Secondly, commercial interests come into play in gauging usage-advertising space on web sites is sold on the basis of readership. Still on this theme, if commercial providers (such as newspapers) have a clearer idea of who is looking at their product they can tailor it to capture a larger readership. On discovering, for example, that a large proportion of its readers were coming in from the United States, The Independent newspaper has begun to emphasize news items that cater to this market.

Looking at the minimal research that has been undertaken, Jones et al [4] estimated use of a medical kiosk by questionnaire only and did not analyze log files. A later study of Healthpoint kiosks by Naven et al [5] did analyze logs of a limited number of users and showed that although only 65 search "episodes" were logged, CCTV (Closed Circuit Television) video showed that the system was actually used by a total of 116 users. The discrepancy was due to users taking over the kiosk before it timed out, thus appearing on the log file to be a continuation of the previous searcher. Also, Jones et al [6] in a comparative study of information technology delivery systems for patients, used log statistics to estimate session times, although the methodological problems associated with this metric were not discussed.

Much of the analysis and development of metrics associated with logs comes from the study of Internet-access (Web site) log files [7,8,9] and OPAC (Online Public Access Catalog) log files [10]. Typically, metrics reported include the number of pages viewed, page view time, number of pages per session, and session length. Early research on Web metrics [11] looked at how to standardize metrics and terminology for the advertising industry. Pitkow [12] noted inconsistencies in terminology and revisited the idea of what terms should be employed to describe the metrics. Neither included an analysis of the problems or an estimate of metric statistics. Chun et al [13] investigated search behavior in a small sample (32 users) by questionnaire and by tracking client machine log files; they identified what they termed search "episodes" but did not clarify the definition of an episode or estimate an episode time. Williamson [14], among others, points to the frustrations posed by logs: "it's a marketer's dream--and worst nightmare: Being able to watch your customers' every move, but possessing only limited tools to influence them." Much of the literature is concerned with the problems and pitfalls associated with Web site log analysis. Zawitz [15] makes the very important point that server logs and their measures were designed originally to measure and manage server traffic and not to analyze the use/effectiveness of Web sites. As a result measures are often misquoted or misunderstood.

### Aims and objectives of The Digital Health Information Project

The Digital Health information project is a far-reaching UK Department of Health funded study into the developing use of digital consumer-health-information services, which is being undertaken by City University in cooperation with Intouch with Health, a leading UK consumer health-information company. Intouch with Health has been responsible for deploying 70 health-information touch screen kiosks around the country, and has a comparable health Web site SurgeryDoor (www.surgerydoor.co.uk). Intouch with Health has made transaction log data from both kiosks and the SurgeryDoor Web site available as a national test-bed against which to benchmark the progress and impact of digital information provision.

The aims of the Digital Health information project are to develop a context-specific understanding of the extent to which and way in which the public interact with the digital delivery of health care information and to examine the wider issues involved: eg, impact of information and communication technologies (ICTs) on the health care profession in general, implications for training needs, and health-inequalities issues.

The paper presented here is part of the Digital Health information project and compares metrics derived from the access logs of the SurgeryDoor Web site with metrics derived from four of Intouch with Health's kiosks. The kiosk sites involved in this study are: the Harpenden general practice surgery, the Edinburgh Royal Infirmary, the Wakefield walk-in health center, and the Esk medical center based in Scotland. Comparisons were made on the basis of data collected for July 2000. During this period the four kiosks recorded an approximate page use of 30,062, and the Web logs recorded an approximate page use of 118,350.

## Methods

As previously mentioned two "platforms," or information-delivery systems, were studied, both the product of the digital health information company Intouch with Health. These were the publicly-accessible Web site SurgeryDoor and a touch screen kiosk.

The purpose of both platforms is to provide the general public (rather than medical professionals) with information about all aspects of health and medical care. This includes advice for people facing a surgical operation, attempting to give up smoking, or simply desirous of leading a healthier lifestyle. For much of the information the text is the same on the two platforms. However, the Web site is more comprehensive in terms of scope of content. It includes, for example, such features as a health-consumer magazine and the latest health news.

**Figure 1:**
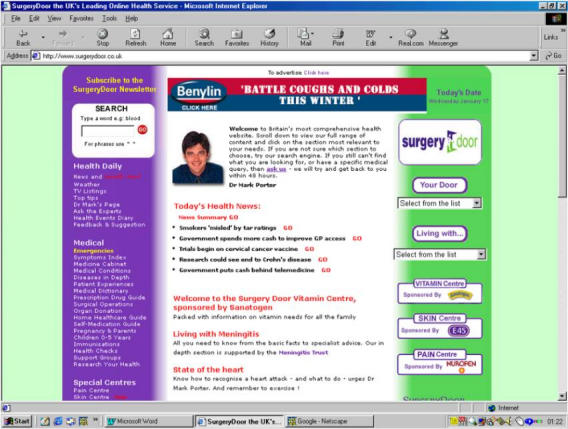
Home page of SurgeryDoor Web site showing menu-hierarchy structure

Both systems are menu-based. The Web site (Figure 1) has menus on both the left and the right of an information page and offers direct access to submenus, with the menu hierarchy listed fully.

**Figure 2:**
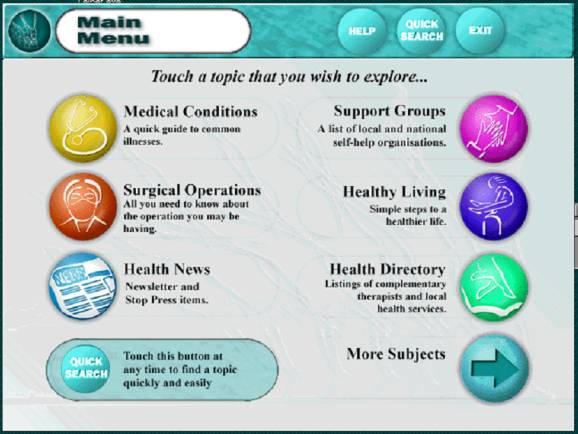
One of two "home page" screens from an Intouch with Health kiosk

Kiosks (Figure 2) have a screen for a set of menus that lead to an information page. The kiosk "home page" consists of eight menu buttons distributed between 2 screens. Accessing all 8 menu-buttons requires "toggling" (switching) between 2 screens. The menu buttons lead to submenu pages.

The menu options for the two platforms are different, but there is some overlap. Both platforms have a Healthy Living menu item and both include sections on the National Health Service (NHS): called NHS & benefits on the Web site, and A-Z of the NHS on the kiosk. The Web site has entries that are not on the kiosk: Community & Fun, Complementary Medicine, and Shopping.

When comparing the platforms, it is important to distinguish between differences in content and differences in structure. Differences in content of the two platforms: there is material on the Web site that is not on the kiosk. Differences in structure of the two platforms: differences concerned with, for example, Medical Conditions and Surgical Operations are principally differences in structure.

The structural differences between the two platforms can be illustrated by the example of Surgical Operations. Although Surgical Operations is not a main heading on the Web site, unlike on the kiosk, it is nevertheless an entry, subsumed under the main heading of Medical. Selecting the Surgical Operations submenu link on the Web site or the Surgical Operations link on the kiosk, gives access to virtually the same content, but via different routes.

- **Web site**
  the Surgical Operations submenu link leads to a page displaying each letter of the alphabet. Selecting a letter-link leads to a list of medical conditions that start with the selected letter. Selecting a medical condition leads to information on the selected condition.
- **Kiosk**
  selecting the Surgical Operations option leads to a main-menu page listing options, eg, Blood vessel systems, Bones, Joints and tendons, Breast, and Children's operations. Selecting an option leads to a comprehensive scrollable alphabetical list of conditions and then to information on the conditions.

However, importantly, the list of conditions on the Web site appears to be identical to the list of conditions on the kiosk and the information for a condition on the Web site appears to be identical to the information for the same condition on the kiosk (on both platforms, the information is under the headings: What is it?, The Operation, Any Alternatives, Before the operation, After - In Hospital, After - At Home, Possible Complications, and General Advice).

Another difference between the two platforms is that the Web site does not collect personal information. The Web site does not ask for age or gender information. Cookies (files or parts of files stored on a Web-site-user's computer, created and subsequently read by a Web site server, containing personal information such as an identification code) could have been used to collect some user information but they were not used on this Web site. The kiosk, however, did prompt users to give their age and gender.

### What are log files?

Log files are machine-generated records of user activity. Both kiosk logs and Internet-access (Web site) logs record user page requests.

### Kiosk log files

Table 1 shows an example of information from a log file of a kiosk user session.

**Table 1:** Example of Information from a Log File of a Kiosk User Session

H	10-Jan-1999	Sun	15:14:17	0000	_Male_ 1 under 15
D	10-Jan-1999	Sun	15:14:18	0001	1###########################001#XXX
D	10-Jan-1999	Sun	15:14:26	0008	#6##########################002#XXX
D	10-Jan-1999	Sun	15:14:32	0014	#6a0#########00001##########003#XXX
D	10-Jan-1999	Sun	15:14:50	0032	#6a2#########00001##########004#XXX
D	10-Jan-1999	Sun	15:15:10	0052	#3-################600001###005#XXX
D	10-Jan-1999	Sun	15:15:14	0056	#3--####0015#######600001###006#XXX
D	10-Jan-1999	Sun	15:15:21	0064	#3--a###0015#00090#600001###007#XXX
T	10-Jan-1999	Sun	15:15:43	0085

- **First column**
  codes page information: H indicates a beginning of a session, D a successful page view, and T a termination sequence generated by the user.
- **Next three columns**
  record the date, day, and time.
- **Column starting 0000**
  records the seconds from the start of a session; this system does not record the time taken by the user to fill in age and gender details; recording of time starts when the user selects "continue" from the age-and-gender page. In the second row, 0001 is the time taken to download the first menu page. This user spent 7 seconds negotiating the first menu page. As shown in the last row, this user session lasted 85 seconds. The longest page view was 21 seconds (calculated by subtracting 64 from 85) and the shortest was 4 seconds (calculated by subtracting 52 from 56). Information in this column will be affected by the kiosk's automatic termination of a session after two minutes of inactivity.
- **Last column**
  records gender and age, in the first row, and page information, in other rows. In the first row, the "1" to the right of Male is the age grouping and repeats (codes) the "under 15" information. In other rows, the numbers and hash signs (#s) relate to page identification codes. The 001 near the end of the line in the second row is a page counter; each line of a log refers to a page viewed by the user. The counter does not record the opening dialogue page where the user records age and gender.

### Web log files

Web log files record a range of information similar to the information in a kiosk log file, but the information collected will depend on the software used and how the server was configured. A Web site page is made up of one or more graphic/text files that are delivered separately and then combined on the client's machine. The SurgeryDoor Web site used Microsoft Internet Information Server 4.0 software that was configured to record requests of files rather than requests of pages.

Table 2 shows an example of information from the SurgeryDoor Web site log.

- **First and second columns**
  record the date and time.
- **"IP number" column**
  records the user's identifying IP (Internet Protocol) number.
- **"Request" column**
  records the user's request.
- **"File request" column**
  records the name and directory of the file downloaded; in the first line, the file requested is "chickenpox.htm" and in the second line the file requested is a graphic file "tv_surgery.jpg."
- **Next two columns**
  record the status of the delivery and the browser compatibility (information on the type of browser software used to access the Web site).
- **Last column**
  records the Web page the user came from.

**Table 2:** Example of Information from a Web Log File

Date	Time	IP number	Request	File request	Delivery Status	Browser compatibility	
2000-04-02	07:58:43	62.252.100.17	GET	/homehealthcareguide/chickenpox.htm	200	Mozilla/4.0+(compatible;+MSIE+4.01;+Windows+95;+VNIE4)	http://www.surgerydoor.co.uk/frame/search.asp?SearchWhere=ALL
2000-04-02	07:58:46	62.252.100.17	GET	/homehealthcareguide/tv_surgery.jpg	304	Mozilla/4.0+(compatible;+MSIE+4.01;+Windows+95;+VNIE4)	-
2000-04-02	07:58:46	212.140.119.160	GET	/images/middle.jpg	200	Mozilla/4.0+(compatible;+MSIE+5.0;+Windows+95;+DigExt)	http://www.surgerydoor.co.uk/frame/topleft.htm

### Differences between kiosk and Web log files

There are a number of important differences between the kiosk and Web log files.

- **User identification**
  Kiosk logs do not provide a user identification number. Web logs provide an IP number. The IP number cannot be traced back to an individual, only to a machine. The extensive use of proxy servers and Point-to-Point Protocol (PPP) connections mean that the IP address might not relate to a specific machine (since the IP address might have been temporarily allocated to that machine) and might relate to a group of users (rather than to an individual). Cookies, which sit on the client's machine, can be employed to help overcome these problems. However Web users may be sensitive to having cookies placed on their machines. Still, Web providers can, and many do, place cookies on client machines, since most browsers are installed by default with cookie acceptance turned on and the average user probably does not turn cookie acceptance off. Cookies were not used on the SurgeryDoor Web site.
- **Multiple users**
  While only one user can use a kiosk at a time, many users can be logged on to a Web site at the same time. Kiosk logs record the consecutive pages viewed by one user. For Web logs, however, the server may have a large number of remote clients logged on simultaneously. The server records a time sequence of file downloads from these clients (that is, the sequence is ordered by the time a file is sent, not by client IP number), so sequences within individual user sessions are identifiable only after the file has been sorted by IP number and, within IP number, by time.
- **Data record**
  Kiosk logs record pages viewed, while Web logs generally record files requested, though the software can be configured to record pages only. As a result it is not uncommon to discard 85% of Web log lines, relating to images downloaded, in a multistage process to estimate pages viewed. Furthermore, as HTML has developed identifying files to reject has become more and more complicated.
- **Time measurement**
  Kiosk logs record the log-off time of the user, either as a result of a user-generated termination request or the automatic log off that happens after two minutes of inactivity. In most cases as far as Web site logs are concerned people do not log off from the Web, they depart anonymously. Typically, a log off or session end is assumed to occur after a specified time of inactivity. The industry (for example, Zawitz [15]) normally assumes a 30-minute inactivity as a termination signal. A 30-minute time out signal is probably too generous (and inaccurate) given a typical page reading time of a minute.

Table 3 shows the metrics that can be generated solely from Internet and kiosk log files. Metrics common to both include: number of pages viewed, number of user sessions, length of session, page view time, number of pages viewed in a session, and subject viewed. Time-based Internet variables have to be calculated on the basis of the lapse in time between the downloading of one page and the downloading of the next page or on the change of a session (as demonstrated by a change in IP address). Individuals may be tracked on the kiosk only if they were asked to log in using an identification name-and this was not the case in our study.

**Table 3:** Metrics that can be generated solely from Internet and kiosk log files

	Internet	Kiosk
Number of pages viewed	&	&
Number of users	&	
Number of user sessions	&	&
Length of session	&	&
Page view time	&	&
Number of pages viewed in a session	&	&
Amount of use per user	&	
Returnees	&	
Geographical location	&	
User gender		&
Subject viewed	&	&
User age		&

### Problems comparing kiosk and Web log files

Comparing "hits" or page impressions (the number of times a Web page has been accessed) between on-line systems poses many problems. The most severe problem is caching of pages when using the Web. Caching of files takes place as the files are downloaded to the client's machine; a file may be cached by the client's machine, the client's provider, or by a user wishing to cache the contents of a Web site to display elsewhere.

Local caching to the client's machine occurs once a page is viewed. Files related to that page are stored on the client's computer; further views of that page are made from this cache and are not recorded in the Web log files. Local caching may be switched off by the client but rarely is, because caching speeds up the reading and access of pages. Hence Internet log files will underreport pages viewed by the number of pages extracted from the cache. Fieber [16] compared videotaped user sessions with the data recorded in the log and found that, depending on the length of the session, between 32% and 55% of transactions were cached and as a result were not recorded by the Web log. This is not an issue with kiosks as their logs record every page viewed by the user. Hence, although estimates of page impressions can be derived for both Internet and kiosk information retrieval systems, the estimates are not strictly comparable. Internet metrics assume the presence of caching and an adjustment cannot easily be made to estimates of page impressions or even to the number of pages used in a session. Browsers can be configured to check for cached pages; however this is unlikely to happen as this slows the delivery of pages-and page-delivery times are a key performance measure for most Web sites.

It sometimes happens that a user will cache the contents of a Web site to deliver the content to a third party or to a population of users; this eases data transfer problems, because information can be delivered locally. The initial and subsequent caching is recorded in the logs of the originating information holder, but page use and hits recorded against the caching server are not. Internet statistics underreport usage because of this.

Robots are another feature of the Internet environment that create havoc with the Internet metrics but are not a feature of kiosk use. Robots are electronic agents used by search engines and organizations to put information about Web page addresses and content in databases. Robot activity is recorded in the log file. Gutzman [9] states that it is estimated that as much as a third of all Web site traffic is made up of robots and spiders (a term that often means robots, as defined in this paper, but which may also mean programs looking for e-mail addresses). Robot use should be excluded from the count of page impressions and many of the software packages available for analyzing log files have an option to exclude robots. Robots can be identified by analyzing IP addresses or by seeing which users visit the "robot.txt" file. This file resides on the host Web server and is accessed by robots. However robots can be set up to not visit the robot.txt file and may have an address that may not be resolved to a domain name server (a domain name server has a database of host computers and their IP addresses). These undeclared robots will be difficult to exclude from the count of page impressions. This makes comparisons between a kiosk and the Internet based on a page-count metric unreliable.

## Results

### Page view time comparison

Page view time appears on the surface to be a metric that can be used to compare kiosk and Internet use. Arguably, view time can be taken as a measure of user satisfaction. Table 4 compares estimates of page view time obtained from kiosk and Internet. Both the frequency distribution of kiosk page view time and Internet page view time were found not to be normally distributed but to be skewed (nonsymmetrical). This is indicated in Table 4 by the differences between the arithmetic mean and the median. The arithmetic mean will be biased and cannot be relied upon if the underlying distribution departs from the normal distribution. To accommodate the departure from the normal distribution the robust estimators (estimators that are not very sensitive to the presence of anomalous values in the sample) the 5% trimmed mean and Huber's M-estimator were generated. Both give estimates of the mean that are not sensitive to the underlying frequency distribution and give unbiased estimates of the mean. The 5% trimmed mean does this by discarding the lowest and highest 2.5% of the values and then computing the mean of the remaining values, Huber's M-estimator is a weighted mean estimate where extreme values are given less weight.

**Table 4:** Page view time in seconds: Kiosk and Internet

	Estimate of kiosk page view time	Estimate of Internet page view time
Mean	27.66	1137.30
Median	10.00	59.00
5% trimmed mean*	17.91	283.82
Huber's M-estimator*	11.19	68.99

^*^ Estimators of the mean that are not very sensitive to the presence of anomalous values in the sample.

Kiosk page view time is less than that recorded for the Web. Given the severity of the departure, as indicated by the difference between the arithmetic mean and the median, from the normal distribution it was decided to use Huber's M-estimator. The mean view time of a kiosk page was approximately 11 seconds and this compares to a mean view time of approximately 69 seconds of an Internet page. Thus, Internet page view time is estimated to be about 6 times that of kiosk page view time-a large difference. There are a number of factors that might explain this, the three most important being:

- **Load up time**
  Internet users are subject to a download waiting time while the server delivers the page to and displays the page on the client's computer. Load up time is likely to be increased by increased use of graphics.
- **Information density**
  The density of information may affect delivery time, and it may be expected that increasing the density of information on the screen will increase the download time.
- **Caching**
  Internet page view time will include the viewing of cached pages. Page view time is the difference between time stamps. However, since logs do not record access to locally-cached pages the time difference will include views of cached pages, thus extending page view time significantly.

Caching is the most influential of the 3 factors since depending on how the Web site is constructed more than half the pages viewed may be from the client's cache. Clearly the more pages that are cached the longer the between-page download time recorded by the server will be. Further, even cached pages are subject to a delay in appearing on the screen.

### Session view time

Session view time also appears to be a worthy metric for comparisons. Longer sessions might indicate greater user satisfaction. Table 5 compares estimates of kiosk and Internet session time. An Internet session end signal was recorded if the user remained on a page for longer than 300 seconds. Session time distributions were skewed and robust estimators were again generated.

**Table 5:** Session view time in seconds: Kiosk and Internet

	Estimate of kiosk Session view time	Estimate of Internet session view time
Mean	201.72	3472.37
Median	123	495
5% trimmed mean	162.87	1936.90
Huber's M-estimator	133.52	586.23

Again Huber's M-estimator is used because of the severity of the departure from the normal distribution. The estimated length of a session at the Web site is approximately 4 times that of a kiosk session-slightly less than 10 minutes for the Web site compared to slightly more than 2 minutes for the kiosk. Load up time will again be a major reason for this difference. Another factor might be Web site design.

### Number of sessions

The number of sessions conducted is a metric common to both kiosks and the Internet. The total number of Internet sessions for July 2000 was estimated to be 34,243. The four kiosks recorded an estimated 2,689 user sessions. To enhance the metric it was decided to estimate the average number of sessions per hour. By using a rate per hour the metric is not sensitive to kiosk opening-hour differences. The overall estimates of user sessions per hour are shown in Table 6.

**Table 6:** Average Number of Sessions Per Hour: Kiosk and Internet

	Estimate of Kiosk sessions per hour	Estimate of Internet sessions per hour
Mean	1.67	46.02
Median	1.65	59.91
5% trimmed mean	1.57	46.07
Huber's M-Estimate	1.48	51.53

The average number of sessions per hour for the kiosks was estimated at about 1.67 (for the kiosk data there is little difference between the mean and median so the mean is used here). The average number of sessions per hour for the Internet is about 51 (for the Internet data there is a difference between the mean and the median so the Huber's M-estimator is used here). Using number of user sessions per hour as a metric we can argue that the Web site provides approximately the same information service as about 30 kiosks. However, as a metric, number of user sessions per hour is only of limited value. The metric gives only a basic comparison and no estimate of user satisfaction or any indication that the user has made use of the information.

### Use per session

In an attempt to make more meaningful statements about the extent to which people use a system, we classified users according to whether they reached only menu (navigation) pages or whether they penetrated to (reached) a page with actual information (non-navigation) content. For what we consider actual use to have occurred, the information seeker has to navigate beyond the collection of initial menu screens and reach the actual information pages. This type of classification is especially important in menu-based systems where the user has to navigate through a number of menu screens to arrive at an information page. This idea can be developed into a manageable and versatile metric by grouping users by the number of pages they have viewed. However, the number of pages that a user has to navigate before reaching an information page is different for the Web and the kiosk, and will be affected by the caching of pages.

- **Web site**
  users recording a single page download were classified as not penetrating to an information page. This classification is based on the Web site as of July 2000. In July 2000, content pages were single HTML pages containing information on a number of topics with a menu of internal links at the top of the page. There were up to 2 higher-level menus. Also, there were a variety of links from the opening page that went directly to an information page. Depending on how users entered the Web site it was highly likely that they would have cached a multiple-topic information page and a menu page by downloading just two pages. The user could then read about related topics by accessing the cached information and menu pages; during this access the server would not record any more hits or page downloads.
- **Kiosk**
  users viewing 4 (or fewer) screens were classified as not having penetrated to an information page. This classification is based on the need to navigate 4 menu screens (see Methods, above) to reach an information page.

Table 7 shows the result of these classifications.

**Table 7:** Percentage of Users Penetrating to Information Pages

User classification*	Kiosk users %	Internet users %
Not penetrating to (reaching) an information page	28.9	34.5
Penetrating to (reaching) an information page	71.1	65.5

^*^ Kiosk users viewing 4 (or fewer) screens were classified as not having penetrated to an information page. Web site users recording a single page download were classified as not penetrating to an information page.

From Table 7 there appears to be slightly more penetration of pages on a kiosk compared to the Internet. Approximately 71% of kiosk users reached an information-rich page compared to an estimated 65.5% on the Internet. This is a metric needing further research; in particular, more research is needed on how users navigate to an Internet information-content page.

## Discussion

None of the metrics examined can be regarded as an effective way of comparing the use of the two different platforms. The most reliable measure, the number of user sessions per hour, is the weakest in terms of understanding obtained. The measure of page penetration, while more informative, needs much more work done on it. Session length is also a promising metric although in regard to the Internet it needs to be adjusted for download time and Web site design if this should prove to be factor. Measures based on the amount of page use and page view time are not comparable as Internet based measures include a significant but non-quantifiable cached element. Internet logs are not easily comparable to kiosk logs.

## Abbreviations

CCTV: Closed Circuit Television
ICT: Information and Communication Technology
IP: Internet Protocol
NHS: National Health Service
OPAC: Online Public Access Catalog
PPP: Point-to-Point Protocol
WAP: Wireless Application Protocol
